# Difference Between Cardiopulmonary Bypass Time and Aortic Cross-Clamping Time as a Predictor of Complications After Coronary Artery Bypass Grafting

**DOI:** 10.21470/1678-9741-2023-0104

**Published:** 2024-02-06

**Authors:** Fabiano Gonçalves Jucá, Fabiane Letícia de Freitas, Maxim Goncharov, Daniella de Lima Pes, Maria Eduarda Coimbra Jucá, Luis Roberto Palma Dallan, Luiz Augusto Ferreira Lisboa, Fábio B. Jatene, Omar Asdrúbal Vilca Mejia

**Affiliations:** 1 Department of Cardiovascular Surgery, Hospital de Messejana Dr. Carlos Alberto Studart Gomes, Fortaleza, Ceará, Brazil; 2 Department of Cardiovascular Surgery, Instituto do Coração, Hospital das Clínicas, Faculdade de Medicina, Universidade de São Paulo, São Paulo, São Paulo, Brazil; 3 Instituto de Pesquisa Hcor, Hospital do Coração, São Paulo, São Paulo, Brazil; 4 Faculdade de Medicina, Unichristus, Fortaleza, Ceará, Brazil

**Keywords:** Coronary Artery Bypass, Cardiopulmonary Bypass, Reoperation, Risk Assessment, Severity of Illness Index, Treatment Outcome

## Abstract

**Introduction:**

Along with cardiopulmonary bypass time, aortic cross-clamping time is directly related to the risk of complications after heart surgery. The influence of the time difference between cardiopulmonary bypass and cross-clamping times (TDC-C) remains poorly understood.

**Objective:**

To assess the impact of cardiopulmonary bypass time in relation to cross-clamping time on immediate results after coronary artery bypass grafting in the Registro Paulista de Cirurgia Cardiovascular (REPLICCAR) II.

**Methods:**

Analysis of 3,090 patients included in REPLICCAR II database was performed. The Society of Thoracic Surgeons outcomes were evaluated (mortality, kidney failure, deep wound infection, reoperation, cerebrovascular accident, and prolonged ventilation time). A cutoff point was adopted, from which the increase of this difference would affect each outcome.

**Results:**

After a cutoff point determination, all patients were divided into Group 1 (cardiopulmonary bypass time < 140 min., TDC-C < 30 min.), Group 2 (cardiopulmonary bypass time < 140 min., TDC-C > 30 min.), Group 3 (cardiopulmonary bypass time > 140 min., TDC-C < 30 min.), and Group 4 (cardiopulmonary bypass time > 140 min., TDC-C > 30 min.). After univariate logistic regression, Group 2 showed significant association with reoperation (odds ratio: 1.64, 95% confidence interval: 1.01-2.66), stroke (odds ratio: 3.85, 95% confidence interval: 1.99-7.63), kidney failure (odds ratio: 1.90, 95% confidence interval: 1.32-2.74), and in-hospital mortality (odds ratio: 2.17, 95% confidence interval: 1.30-3.60).

**Conclusion:**

TDC-C serves as a predictive factor for complications following coronary artery bypass grafting. We strongly recommend that future studies incorporate this metric to improve the prediction of complications.

## INTRODUCTION

Cardiopulmonary bypass time (CPBT), together with prolonged aortic cross-clamping time, is associated with increased intra and postoperative complications after cardiac surgery^[1-4]^. Those complications, caused by myocardial damage and the increased inflammatory response, can lead to low cardiac output syndrome, renal dysfunction, vasoplegia, neurological deficit, and increased ventilation time^[5,6]^.

There is no consensus in the literature regarding the ideal time that leads to greater surgical safety. However, decreasing aortic cross-clamping time and CPBT is one of the most challenging issues in cardiac surgery^[1,2]^. Moreover, the patients’ clinical profile, often associated with prolonged surgery, make it difficult to understand what really affects the results of increasingly challenging procedures.

A study conducted by Ruggieri et al.^[1]^ showed that aortic cross-clamping time was related to risk of mortality, atrial fibrillation, prolonged intensive care unit stay, and incidence of major adverse cardiac and cerebrovascular events. Nevertheless, the traditional risk scores used do not consider intraoperative variables, let alone the time difference between cardiopulmonary bypass (CPB) and aortic cross-clamping times (TDC-C).

In this regard, Al-Sarraf et al.^[7]^ performed a study that analyzed lowand high-risk patients undergoing all types of cardiac surgery. The study concluded that both groups, lowand high-risk, had higher incidences of morbidity and mortality observed in patients with prolonged aortic cross-clamping time.

Special attention should be paid to TDC-C. The association of this parameter with post-surgical outcomes remains an area of interest that requires further research, prompting the authors to evaluate it in this study.

It is necessary to emphasize the current need for risk assessment of morbidity and mortality before and after cardiac surgery, since one third of the perioperative events that lead to patient’s death occur in the operating room^[8,9]^. Therefore, for better predictability regarding potential complications after surgery, the surgical risk stratification should always be updated according to the patient’s evolution.

CPBT reflects the complexity of the surgery itself along with technical difficulties in performing the planned surgery due to unfavorable anatomy or intraoperative complications, which can increase the planned time^[10]^. In turn, an increased TDC-C usually indicates intraoperative complications, that require longer CPB duration after removal of the aortic clamp. Therefore, it seems more logical to think that increased TDC-C would be more related to complications than increased CPBT.

Therefore, the aim of this study was to assess the association of CPBT and TDC-C with complications after coronary artery bypass grafting (CABG).

## METHODS

This study is a subanalysis of the Registro Paulista de Cirurgia Cardiovascular (REPLICCAR) II database. REPLICCAR II was a prospective, observational, multicenter study that included five centers in the state of São Paulo, Brazil. Patients were operated on consecutively, from July 2017 to June 2019.

The REPLICCAR II database^[11]^ has patients aged ≥ 18 years who underwent elective or urgent primary isolated CABG. The platform for data collection was created in REDCap (http://www.project-redcap.org) especially for the project. Data collection was made online, and the database contains the same variables and definitions as the Society of Thoracic Surgeons (STS) collection system version 2.9.

Due to the type of study, the patients’ clinical profile, as well as surgery complexity, were not adjusted. Patients who underwent emergency surgery, off-pump surgery, or died in the operating room were not included in this analysis.

Through univariate logistic regression, cutoff point was determined as 30 minutes in TDC-C and 140 minutes in CPBT.

The primary outcome of this study is in-hospital mortality. Secondary outcomes were reoperation, cerebrovascular accident (CVA), acute kidney failure, prolonged ventilation time, and surgical wound infection.

### Definition of Groups

For a better understanding, four groups were created based on the CPBT and TDC-C cohort levels related to the increase in complications after CABG.

The definition of the groups was carried out as follows:

Group 1: CPBT < 140 minutes and TDC-C < 30 minutes.
Group 2: CPBT < 140 minutes and TDC-C > 30 minutes.
Group 3: CPBT > 140 minutes and TDC-C < 30 minutes.
Group 4 CPBT > 140 minutes and TDC-C > 30 minutes.

### Statistical Analysis

R software version 4.0.2 was used to perform statistical analysis. In the descriptive analysis, continuous variables were expressed as mean and standard deviation, and asymmetric continuous variables were described through median and interquartile range (IQR), while categorical variables were expressed in terms of frequencies and percentages. Categorical independent variables and outcomes were analyzed by comparing proportions using chi-square or Fisher’s exact test, as appropriate. Continuous independent variables and outcomes were evaluated by comparing the means using Kruskal-Wallis test.

For the definition of the cutoff point, a univariate logistic regression of the outcomes (primary and secondary) was performed on the CPBT and TDC-C; it was defined when the time obtained a relative risk referring to most of the outcome variables.

All outcomes were analyzed using univariate logistic regression to evaluate the odds ratio (OR) and the performance of the four groups. The OR and the 95% confidence interval (CI) were expressed. *P*-values < 0.05 were considered significant.

### Ethics and Informed Consent

The current study is a subanalysis of the REPLICCAR II project, approved by the Research Ethics Committee (CAPPesq) of the Hospital das Clínicas of the Universidade de São Paulo, opinion number 5,603,742, under CAAE registration number 66919417.6.1001.0068 and SDC number 4506/17/006. Informed consent was waived due to the study design (the study used in-hospital information system).

## RESULTS

The study evaluated 3,090 patients who underwent CABG. The median age was 63 (57-70) years, 25.79% were females, and 19.16% of patients had an urgent admission status. The mean surgery time was 4.52±1.43 hours, and the mean CPBT and aortic cross-clamping time were 76.78±27.53 minutes and 58.22±23.36 minutes, respectively. The TDC-C was 18.56 ±12.0 minutes.

Table 1 shows the characteristics of the four groups evaluated.

**Table 1:** Patients’ characteristics (REPLICCAR II, São Paulo, Brazil, 2023).

Characteristics	Total		Group 1 (n=2596)		Group 2 (n=416)		Group 3 (n=32)		Group 4 (n=46)		*P*-value
	n	%	n	%	n	%	n	%	n	%	
Age (years)^*^	63 (57-70)		63 (57-69)		64 (57-70)		62.5 (56.75-69.25)		66 (60-70)		0.41
Gender (female)	797	25.79	687	26.46	95	22.84	8	25.00	7	15.22	0.16
Urgent admission	592	19.16	481	18.53	84	20.19	15	46.88	12	26.09	0.001
Body mass index											
< 18.5	34	1.10	29	1.12	5	1.20	0	0.00	0	0.00	
18.5-24.9	906	29.32	762	29.35	124	29.81	8	25.00	12	26.09	0.81
25-29.9	780	25.24	669	25.77	94	22.60	8	25.00	9	19.57	
≥ 30	1370	44.34	1136	43.76	193	46.39	16	50.00	25	54.35	
Previous myocardial infarction	1616	52.30	1368	52.70	206	49.52	14	43.75	28	60.87	0.45
Systemic arterial hypertension	2699	87.35	2268	87.37	370	88.94	28	87.50	33	71.74	0.02
Diabetes mellitus	1511	48.90	1251	48.19	219	52.64	20	62.50	21	45.65	0.14
Cerebrovascular disease^^1^^	280	9.06	226	8.71	45	10.82	3	9.38	6	13.04	< 0.001
Atrial fibrillation	47	1.52	40	1.54	4	0.96	3	9.38	0	0.00	-
Ejection fraction < 30%	48	1.55	34	1.31	12	2.88	2	6.25	0	0.00	-
Kidney failure	204	6.60	161	6.20	31	7.45	5	15.63	7	15.22	< 0.001
CCS angina classification											
4	302	9.77	252	9.71	41	9.86	3	9.38	6	13.04	0.83
NYHA classification											
I and II	2692	87.12	2272	87.52	352	84.62	29	90.63	39	84.78	0.03
III and IV	398	12.88	324	12.48	64	15.38	3	9.38	7	15.22	
STS score (mortality)^*^	0.64 (0.42-1.01)		0.63 (0.41-0.99)		0.65 (0.43-1.09)		0.78 (0.40-1.49)		0.74 (0.54-1.33)		0.04
Intraoperative period											
Surgery time (hours)^**^	4.52 1.43		4.43±1.43		4.85±1.26		6.64±1		6.28±1.13		< 0.001
Cardiopulmonary bypass time (minutes)^**^	76.78±27.53		71.06±23.3		97.51±18.81		149.84±10.81		161.04±21.65		< 0.001
Aortic cross-clamping time (minutes)^**^	58.22±23.36		56.5±22.1		58.49±17.11		130.53±11.48		102.52±24.53		< 0.001
TDC-C (minutes)^**^	18.56±12.07		14.56±5.93		39.02±9		19.31±6.66		58.52±27.74		< 0.001
Postoperative period											
Reoperation	114	3.69	85	3.27	22	5.29	3	9.38	4	8.70	0.01
CVA	41	1.33	23	0.89	14	3.37	3	9.38	1	2.17	< 0.001
Kidney failure	193	6.25	141	5.43	41	9.86	3	9.38	8	17.39	< 0.001
Prolonged ventilation (> 24 hours)	39	1.26	32	1.23	3	0.72	2	6.25	2	4.35	0.02
Surgical wound infection	67	2.17	53	2.04	12	2.88	1	3.13	1	2.17	0.46
In-hospital mortality	93	3.01	62	2.39	21	5.05	3	9.38	7	15.22	< 0.001

CCS=Canadian Cardiovascular Society; CVA=cerebrovascular accident; NYHA=New York Heart Association; REPLICCAR=Registro Paulista de Cirurgia Cardiovascular; STS=Society of Thoracic Surgeons; TDC-C=time difference between cardiopulmonary bypass and aortic cross-clamping times

^1^Cerebrovascular disease: CVA, transient ischemic attack, or carotid stenosis

^*^Median and interquartile range

^**^Mean and standard deviation

Group 3 had a higher prevalence of urgently admitted patients, representing 46.88% (Group 1: 18.53%; Group 2: 20.19%; Group 4: 26.09%; *P*=0.001); Group 4 had a higher incidence of previous CVA, representing 13.04% of patients (Group 1: 8.71%; Group 2: 10.82%; Group 3: 9.38%; *P*<0.001). Patients in Groups 2 and 3 had similar incidences of previous kidney failure, with 15.63% and 15.22%, respectively (*P*<0.001). Group 4 patients had a higher incidence of Canadian Cardiovascular Society grade 4 angina compared to the other groups, with 13.04% (Group 1: 9.71%; Group 2: 9.86%; Group 3: 9.38%), but with no significant difference (*P*=0.83). Groups 2 and 4 had similar incidences of New York Heart Association functional classes III and IV, with 15.38% and 15.22%, respectively (*P*=0.03). The STS mortality score had the highest median in Group 3 with 0.78% (IQR 0.40-1.49) and Group 4 with 0.74% (IQR 0.54-1.33) (*P*=0.04).

Regarding intraoperative variables, the mean time of surgery was longer in Group 3 (6.64±1.00 hours), close to the time found in Group 4 (6.28±1.13 hours). Groups 1 and 2 had time of surgery similar to the total sample (4.43±1.43 and 4.85±1.26 hours, respectively; *P*<0.001). The mean CPBT was longer in Group 4 (Group 1: 71.06±23.30 minutes; Group 2: 97.51±18.81 minutes; Group 3: 149.84±10.81 minutes; Group 4: 161.04±21.65 minutes; *P*<0.001). Mean aortic cross-clamping time was longer in Group 3 (Group 1: 56.5±22.1 minutes; Group 2: 58.49±17.11 minutes; Group 3: 130.53±11.48 minutes; Group 4: 102.52±24.53 minutes; *P*<0.001). The mean TDC-C was longer in Groups 2 and 4 (39.02±9 and 58.52±27.74 minutes, respectively; *P*<0.001).

As for the outcomes, reoperation was more prevalent in Groups 3 and 4 (9.38% and 8.70%, respectively; *P*=0.01). Postoperative CVA was higher in Group 3, with 9.38% (Group 1: 0.89%; Group 2: 3.37%; Group 4: 2.17%; *P*<0.001). There was a high prevalence of kidney failure in Group 4, with 17.39% of cases (Group 1: 5.43%; Group 2: 9.86%; Group 3: 9.38%; *P*<0.001). Prolonged ventilation (over 24 hours) showed a higher incidence in Group 3, with 6.25% of patients (Group 1: 1.23%; Group 2: 0.72%; Group 4: 4.35%; *P*=0.02). Deaths were more representative in Group 4, occurring in 15.22% of patients (Group 1: 2.93%; Group 2: 5.05%; Group 3: 9.38%; *P*<0.001). There was no significant difference between the groups in terms of surgical wound infection.

### Cutoff Point

The > 140 minutes on CPBT cutoff point showed risk in reoperation (OR: 2.67; 95% CI: 1.20-5.95; *P*=0.01), CVA (OR: 4.34; 95% CI: 1.51-12.50; *P*=0.006), kidney failure (OR: 2.55; 95% CI: 1.32-4.91; *P*=0.005), prolonged ventilation (OR: 4.59; 95% CI: 1.59-13.26; *P*=0.004), and in-hospital mortality (OR: 5.18; 95% CI: 2.58-10.43; *P*<0.001) (Table 2).

**Table 2:** Cutoff point definition in CPBT (REPLICCAR II, São Paulo, Brazil, 2023).

Variable	OR	95% CI	*P*-value
Reoperation	2.67	1.20-5.95	0.01
CVA	4.34	1.51-12.50	0.006
Kidney failure	2.55	1.32-4.91	0.005
Prolonged ventilation	4.59	1.59-13.26	0.004
Surgical wound infection	1.19	0.29-4.96	0.80
In-hospital mortality	5.18	2.58-10.43	< 0.001

CI=confidence interval; CPBT=cardiopulmonary bypass time; CVA=cerebrovascular accident; OR=odds ratio; REPLICCAR=Registro Paulista de Cirurgia Cardiovascular

The > 30 minutes on TDC-C cutoff point showed risk in reoperation (OR: 1.72; 95% CI: 1.09-2.69; *P*=0.02), CVA (OR: 3.35; 95% CI: 1.76-6.39; *P*<0.001), kidney failure (OR: 2.04; 95% CI: 1.45-2.87; *P*<0.001), and in-hospital mortality (OR: 2.54; 95% CI: 1.61-4.00; *P*<0.001) (Table 3).

**Table 3:** Cutoff point definition in TDC-C (REPLICCAR II, São Paulo, Brazil, 2023).

Variable	OR	95% CI	*P*-value
Reoperation	1.72	1.09-2.69	0.02
CVA	3.35	1.76-6.39	< 0.001
Kidney failure	2.04	1.45-2.87	< 0.001
Prolonged ventilation	0.83	0.32-2.14	0.7
Surgical wound infection	1.38	0.74-2.54	0.30
In-hospital mortality	2.54	1.61-4.00	< 0.001

CI=confidence interval; CVA=cerebrovascular accident; OR=odds ratio; REPLICCAR=Registro Paulista de Cirurgia Cardiovascular; TDC-C=time difference between cardiopulmonary bypass and aortic cross-clamping times

### Association of Outcomes with Groups

Group 1 was used as the reference group (Table 4).

**Table 4:** Univariate logistic regression for each outcome and comparison between the groups.

Variable	OR	95% CI	*P*-value
Reoperation			
Group 2	1.64	1.01-2.66	0.02
Group 3	3.05	0.91-10.22	
Group 4	2.81	0.98-8.02	
CVA			
Group 2	3.85	1.99-7.63	< 0.001
Group 3	11.27	3.29-40.69	
Group 4	2.48	0.32-18.80	
Kidney failure			
Group 2	1.90	1.32-2.74	< 0.001
Group 3	1.80	0.54-5.98	
Group 4	3.66	1.67-8.00	
Prolonged ventilation			
Group 2	0.58	0.17-1.90	0.03
Group 3	5.34	1.22-23.30	
Group 4	3.64	0.84-15.67	
Surgical wound infection			
Group 2	1.42	0.75-2.69	0.07
Group 3	1.54	0.21-11.54	
Group 4	1.07	0.14-7.87	
In-hospital mortality			
Group 2	2.17	1.30-3.60	< 0.001
Group 3	4.22	1.25-14.25	
Group 4	7.33	3.15-17.04	

CI=confidence interval; CVA=cerebrovascular accident; OR=odds ratio

Group 2 had a significant association with reoperation (OR: 1.64; 95% CI: 1.01-2.66), CVA (OR: 3.85; 95% CI: 1.99-7.63), kidney failure (OR: 1.90; 95% CI: 1.32-2.74), and in-hospital mortality (OR: 2.17; 95% CI: 1.30-3.60).

Group 3 was significantly associated with CVA (OR: 11.27; 95% CI: 3.29-40.69), prolonged ventilation (OR: 5.34; 95% CI: 1.22-23.30), and in-hospital mortality (OR: 4.22; 95% CI: 1.25-14.25).

Group 4 was significantly associated with kidney failure (OR: 3.66; 95% CI: 1.67-8.00) and in-hospital mortality (OR: 7.33; 95% CI: 3.15-17.04).

## DISCUSSION

It is important to notice that the main interest for our study was Group 2, which had a short CPBT, but at the same time it had prolonged TDC-C. Despite this, we find it relevant to discuss all our findings.

Increased CPBT was associated with mortality within 90 days in the study by Jun Zheng et al.^[12]^. Thus, the decrease in CPBT and TDC-C proved to be beneficial for the patient, as well as in Group 1 (Table 4), which was treated as a reference group for the regression analysis. This reinforces that the decrease in CPBT and TDC-C would be related to fewer complications and in-hospital mortality.

Bucerius et al.^[13]^ identified that CPBT > 2 hours was an independent predictor of CVA, increasing the risk by 1.42 times. CPBT was also an independent predictor of early CVA in 2,972 patients undergoing CABG and/or valve surgery. Aortic cross-clamping time proved to be an independent predictor in the work by Svedjeholm et al.^[14]^, with a significant association with post-surgical neurological events. In the present study, the groups showed significant differences in prediction of CVA. Group 3 with prolonged CBPT showed risk elevation of CVA (OR: 11.27; 95% CI: 3.29-40.69), but also Group 2 with prolonged TDC-C and short CBPT showed elevated risk for stroke (OR: 3.85; 95% CI: 1.99-7.63). Group 4 in that case showed risk elevation as well, but at the same time the CI was too wide (OR: 2.81; 95% CI: 0.32-18.80), which makes it non-significant.

Kidney dysfunction after cardiac surgery remains a common complication and an independent predictor of postoperative morbidity and mortality, which shows the significant association with CPBT^[15]^. In the current study, Groups 2 and 4 with increased TDC-C showed the significant association with postoperative kidney failure (OR: 1.90 and 3.66; 95% CI: 1.32-2.74 and 1.67-8.00, respectively) regardless of whether CPBT was greater or less than 140 minutes.

Studies have shown that prolonged CPB use may increase the risk of prolonged ventilation after surgery^[16]^. In the present study, Group 3 had a 5.34-fold risk of prolonged ventilation (95% CI: 1.22-23.30).

In case of surgical wound infection, none of the groups showed significant association with this postoperative complication.

A 2017 study showed that the increase in CPBT can have unfavorable consequences when > 180 minutes^[17]^. In another study by Salis et al.^[3]^, an increased risk of death of 1.57 times was observed in the group with prolonged CPBT. In turn, the present study showed the same trend for Groups 2, 3, and 4. Group 2 with prolonged TDC-C showed significant risk elevation for death in 2.17 times (95% CI: 1.30-3.60). Group 3 with prolonged CPBT showed higher mortality risk (OR: 4.22; 95% CI: 1.25-14.25). But the greatest impact on in-hospital mortality was exerted by Group 4 with prolonged CPBT and TDC-C (OR: 7.33; 95% CI: 3.15-17.04).

One explanation for these findings is that an increased CPBT can most often be very well conducted, however an increased TDC-C would be related to difficulties in weaning from CPB, which justifies that this is a more reliable variable to show risk of complications.

### Limitations

The current study did not aim to find the risk factors that led to prolonged TDC-C, but to evaluate this parameter as a risk factor. That is why TDC-C was treated as a predictor, but not as an outcome.

This observational study analyzes only in-hospital data, so it still can be prone to confounding factors. Also, there was no patient follow-up, so the database contains only in-hospital outcomes. The study did not evaluate the impact of other potential factors, such as surgeon experience or hospital volume, on the outcomes.

## CONCLUSION

TDC-C serves as a predictive factor for complications following CABG. We strongly recommend that future studies incorporate this metric to improve the prediction of complications.

**Table:** 

Authors’ Roles & Responsibilities	
FGJ	Substantial contributions to the conception or design of the work; or the acquisition, analysis, or interpretation of data for the work; final approval of the version to be published
FLF	Substantial contributions to the conception or design of the work; or the acquisition, analysis, or interpretation of data for the work; agreement to be accountable for all aspects of the work in ensuring that questions related to the accuracy or integrity of any part of the work are appropriately investigated and resolved; final approval of the version to be published
MG	Substantial contributions to the conception or design of the work; or the acquisition, analysis, or interpretation of data for the work; final approval of the version to be published
DLP	Substantial contributions to the conception or design of the work; or the acquisition, analysis, or interpretation of data for the work; final approval of the version to be published
MECJ	Substantial contributions to the conception or design of the work; or the acquisition, analysis, or interpretation of data for the work; final approval of the version to be published
LRPD	Drafting the work or revising it critically for important intellectual content; final approval of the version to be published
LAFL	Drafting the work or revising it critically for important intellectual content; final approval of the version to be published
FBJ	Drafting the work or revising it critically for important intellectual content; final approval of the version to be published
OAVM	Drafting the work or revising it critically for important intellectual content; agreement to be accountable for all aspects of the work in ensuring that questions related to the accuracy or integrity of any part of the work are appropriately investigated and resolved; final approval of the version to be published
